# Process of developing models of maternal nutrition interventions integrated into antenatal care services in Bangladesh, Burkina Faso, Ethiopia and India

**DOI:** 10.1111/mcn.13379

**Published:** 2022-06-14

**Authors:** Tina Sanghvi, Phuong Hong Nguyen, Sebanti Ghosh, Maurice Zafimanjaka, Tamirat Walissa, Robert Karama, Zeba Mahmud, Manisha Tharaney, Jessica Escobar‐Alegria, Elana Landes Dhuse, Sunny S. Kim

**Affiliations:** ^1^ Alive & Thrive Initiative, FHI Solutions Washington District of Columbia USA; ^2^ Poverty, Health and Nutrition Division, International Food Policy Research Institute Washington District of Columbia USA; ^3^ Alive & Thrive Initiative, FHI Solutions New Delhi India; ^4^ Alive & Thrive Initiative, FHI Solutions Ouagadougou Burkina Faso; ^5^ Alive & Thrive Initiative, FHI Solutions Addis Ababa Ethiopia; ^6^ Alive & Thrive Initiative, FHI Solutions Dhaka Bangladesh; ^7^ Alive & Thrive Initiative, FHI Solutions Abidjan Cote d'Ivoire

**Keywords:** ANC, counselling, integrated health services, maternal nutrition, micronutrient supplements, pregnancy weight gain

## Abstract

Integrating nutrition interventions into antenatal care (ANC) requires adapting global recommendations to fit existing health systems and local contexts, but the evidence is limited on the process of tailoring nutrition interventions for health programmes. We developed and integrated maternal nutrition interventions into ANC programmes in Bangladesh, Burkina Faso, Ethiopia and India by conducting studies and assessments, developing new tools and processes and field testing integrated programme models. This paper elucidates how we used information and data to contextualize a package of globally recommended maternal nutrition interventions (micronutrient supplementation, weight gain monitoring, dietary counselling and counselling on breastfeeding) and describes four country‐specific health service delivery models. We developed a Theory of Change to illustrate common barriers and strategies for strengthening nutrition interventions during ANC. We used multiple information sources including situational assessments, formative research, piloting and pretesting results, supply assessments, stakeholder meetings, household and service provider surveys and monitoring data to design models of maternal nutrition interventions. We developed detailed protocols for implementing maternal nutrition interventions; reinforced staff capacity, nutrition counselling, monitoring systems and community engagement processes; and addressed micronutrient supplement supply bottlenecks. Community‐level activities were essential for complementing facility‐based services. Routine monitoring data, rapid assessments and information from intensified supervision were important during the early stages of implementation to improve the feasibility and scalability of models. The lessons from addressing maternal nutrition in ANC may serve as a guide for tackling missed opportunities for nutrition within health services in other contexts.

## Key messages

- Integrating evidence‐based nutrition interventions into ANC to reach PW at scale is urgently needed for improving maternal and newborn health and nutrition.
- The Theory of Change and steps for strengthening nutrition interventions based on four‐country experiences provide practical guidance on addressing missed opportunities for nutrition in ANC.
- Strategic use of data can contextualize global maternal nutrition guidelines, protocols, capacity building and supervision approaches, and improve micronutrient supply chains and record‐keeping as part of health services strengthening.
- Engaging family and community members to support PW and improving the knowledge and self‐confidence of PW are important elements of all country programme models.

## Graphical abstract

This paper describes the process of developing models of maternal nutrition interventions integrated into antenatal care services in Bangladesh, Burkina Faso, Ethiopia and India. We developed a Theory of Change to illustrate common barriers and strategies for strengthening nutrition interventions during antenatal care. We used multiple information sources to contextualize a package of globally recommended maternal nutrition interventions and developed four country‐specific models to tackle missed opportunities for nutrition within health services. Theory of change^1^ for strengthening maternal nutrition interventions^2^ in ANC services.

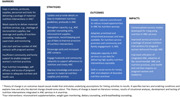

## 1 INTRODUCTION

According to the Global Burden of Disease analysis, there were 135.3 million live births and many more pregnancies in 2019 (GBD Demographics Collaborators, 2020). The outcomes of these pregnancies are substantially related to the nutritional status of mothers during and before pregnancy. Poor maternal nutrition is associated with increased risks of maternal morbidity and higher risks of foetal losses, preterm delivery, growth restriction, stunting and cognitive impairment (Ramakrishnan et al., 2012; Victora et al., 2021). The World Health Organization (WHO) emphasized the importance of addressing maternal undernutrition to accelerate progress in achieving global nutrition targets and Sustainable Development Goals for stunting, anaemia in women, low birthweight and wasting (Christian et al., 2013; WHO, 2021).

During pregnancy, women need to consume adequate energy and good quality protein, carbohydrates, fats, vitamins and minerals (IOM, 2009; Kominiarek & Rajan, 2016). Yet, maternal diets in many low‐ and middle‐income countries (LMICs) are suboptimal with imbalanced macronutrient intakes and inadequate micronutrient content (Lee et al., 2013). To overcome these challenges, various interventions have been suggested including counselling on the use of nutrient‐rich, locally available foods and food and nutrient supplementation (e.g., iron folic acid [IFA], calcium and multiple micronutrients) and weight gain monitoring to maintain healthy weight gain by adjusting food intakes to prevent undernutrition or overweight and obesity (WHO, 2016). Antenatal care (ANC) guidelines published by WHO (2016) highlighted the importance of integrating evidence‐based nutrition interventions. WHO also recommends the preparation of pregnant women (PW) and their family members by ANC providers to place the baby on their chest immediately after delivery, not feeding the baby anything other than colostrum or breast milk and counselling on critical skills to initiate breastfeeding (WHO, 2018).

ANC is one of the most widely used health services and provides an important opportunity to reach PW with a package of nutrition interventions (Heidkamp et al., 2021; Shekar et al., 2021). A study using representative data from 81 LMICs found that high‐quality, evidence‐based, health interventions delivered to mothers and their newborns who are already seeking care would lower maternal deaths by an estimated 28%, neonatal deaths by 28% and stillbirths by 22%, as compared with a scenario without improvement in quality of care (Chou et al., 2019). Despite the WHO recommendations and evidence, large gaps exist and too many PW who attend ANC do not receive nutrition interventions (Heidkamp et al., 2020; Kavle & Landry, 2018).

Integrating globally recommended maternal nutrition interventions (MNIs) into existing large‐scale ANC programmes requires systematic adaptation to ensure relevance and feasibility (Barreix et al., 2020). High‐quality service delivery requires fit‐for‐purpose tools and skills and motivation of ANC providers and community health workers to apply them. Supportive care provided to PW is needed to enable the adoption of recommended nutrition practices. However, there is no documented experience on how to adapt a package of multiple MNIs for integration into ANC. This paper aimed to: (1) elucidate how information and data were used to contextualize a package of MNIs, specifically micronutrient supplementation, weight gain monitoring, dietary counselling and counselling on breastfeeding, for integration into existing ANC services; and (2) describe country‐specific programme models of maternal nutrition services delivered through large‐scale ANC programmes in Bangladesh, Burkina Faso, Ethiopia and India. The MNIs were designed and supported by Alive & Thrive (A&T), an initiative that supports the scaling up of nutrition interventions to save lives, prevent illnesses and contribute to healthy growth and development through improved maternal and infant and young child nutrition practices in several African and Asian countries.

## 2 METHODS

We worked in four countries (Bangladesh, Burkina Faso, Ethiopia and India) alongside government and other stakeholders to design and implement a package of globally recommended MNIs (WHO, 2016) through existing large‐scale ANC programmes. The MNIs include micronutrient supplementation (IFA and calcium distribution and counselling), weight gain monitoring (measurement and counselling), dietary counselling (on meal frequency, food amounts and dietary diversity) and counselling on breastfeeding (early initiation [EIBF] and exclusive breastfeeding [EBF)]. In each country, we incorporated global recommendations into programme protocols, reinforced system readiness to implement the protocols through well‐defined tasks and staff skills, developed and disseminated training tools and job aids, identified gaps, improved equipment/supply chain specifications and strengthened record‐keeping and data‐use protocols.

**Figure 1:**
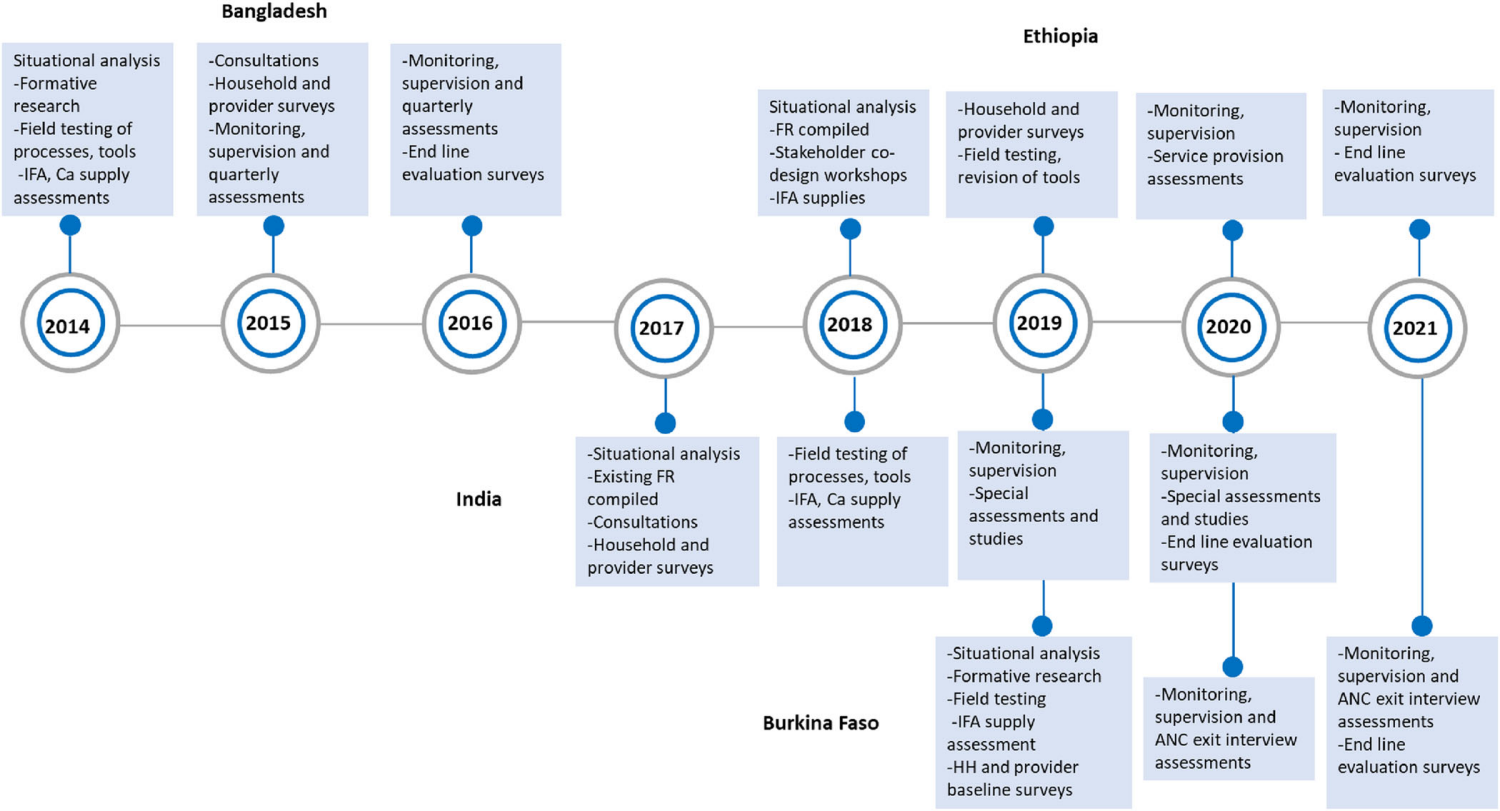
Overview of timeline and main data sources to adapt maternal nutrition interventions for integration into ANCs. ANC, antenatal care; Ca, calcium supplements; FR, formative research; IFA, iron and folic acid supplements; MNIs, package of maternal nutrition interventions.

The duration of intervention planning, development and implementation varied between 3 and 4 years per country, starting in 2014 until ending in 2021 (Figure 1). Programmes in Bangladesh and India were completed in 2016 and in early 2020, respectively, and thus were not affected by the COVID‐19 pandemic. Implementation in Burkina Faso and Ethiopia ended in 2021, and utilization of ANC and other nutrition services decreased temporarily in mid‐2020 due to COVID‐19 restrictions. Several adaptations were applied to maintain and restore services during the pandemic in these countries. Country teams (including the Ministry of Health staff responsible for ANC and nutrition units, community workers and volunteers, national nongovernmental organizations (NGOs), academic institutions, research institutes and A&T staff) were involved in different aspects of design, implementation and assessments. In 2020 and 2021, we compiled our programme implementation documents, protocols and tools to extract information on steps taken to tailor the interventions and strategies to country contexts and to describe each country's model.

**Figure 2:**
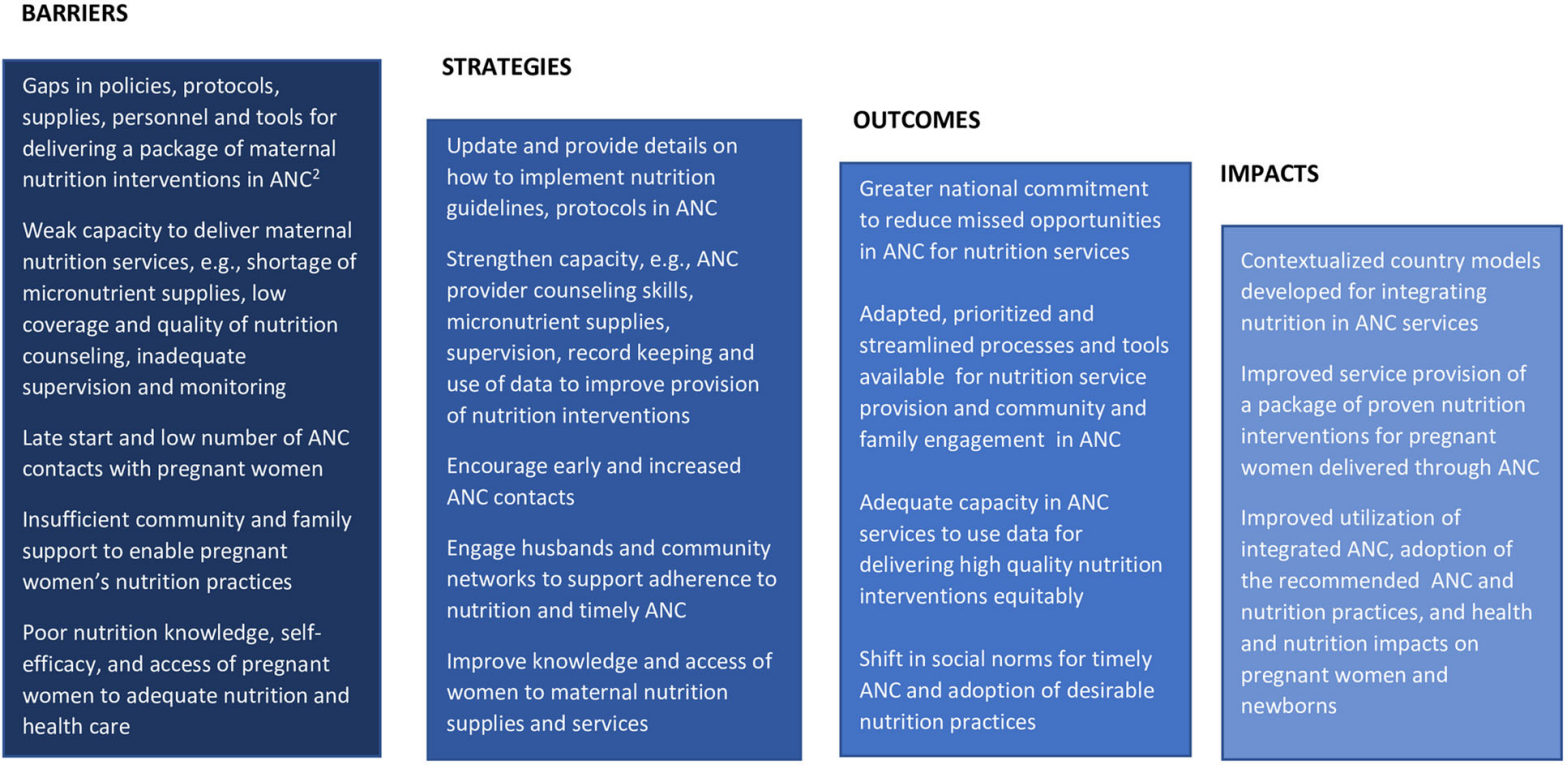
Theory of change^1 ^for strengthening maternal nutrition interventions^2^ in antenatal care services.

First, an overarching Theory of Change that illustrates the country's programme needs was developed to describe common barriers and strategies, expected outcomes and health and nutrition impacts (Figure 2). The Theory of Change was derived from desktop reviews of the literature, situational assessments, special studies and midline assessments, formative studies and surveys conducted by collaborators in the four countries. Strategies for delivering nutrition interventions through ANC were shaped by WHO's recommendations (WHO, 2016), WHO health systems building blocks (WHO, 2007) and the socioecological model of social and behaviour change applied previously by A&T (Sanghvi et al., 2016).

Data and information were used before, during and after the interventions were launched to continuously find gaps and modify the country programme models. In the early stages of implementation, the data and information sources included (1) situational analyses (assessment of policy and protocol gaps and the strengths and weaknesses in ANC service platforms), qualitative research (on perceptions and motivations of PW, family members and community members) and stakeholder workshops convened by government authorities and A&T to discuss policies, feasible protocol modifications, task allocation and engagement of other sectors (such as relevant ministries, local governments and religious institutions); (2) field testing of processes and tools designed to strengthen nutrition interventions; (3) assessments of micronutrient supplement supply chains; (4) baseline surveys with PW and recently delivered women (RDW) as well as their family members, health providers and community health workers. Codesigning workshops were held with ANC service decision‐makers, supervisors and managers, ANC providers, members of local health and development NGOs, village leaders, local government authorities, health volunteers and family members of PW. During the workshops, roles and responsibilities of actors were identified and tasks were allocated where needed. These workshops provided a reality check for the extent to which ANC providers could undertake additional tasks related to MNIs and helped to build ownership among health managers, service providers and community members who were represented in the process. In Ethiopia and India, we used data from existing qualitative research studies to identify motivations and perceptions of health workers and PW (Clemmons & Griffiths, 2016; CMS, 2015; Hirvonen & Wolle, 2019), while new qualitative studies were conducted in Bangladesh and Burkina Faso (Ky‐Zerbo et al., 2019; Schuler, 2015). Structured surveys of PW, RDW, ANC providers and community workers were conducted to generate baseline data for impact evaluation and informed the programme designs (Kim, Ouédraogo, et al., 2020; Kim, Sununtnasuk, et al., 2020, 2020; Nguyen et al., 2015, 2018). Multivariable analysis in Bangladesh and India (Nguyen, Kim, et al., 2017; Nguyen et al., 2019) helped us to prioritize factors associated with maternal nutrition practices, while qualitative research in all countries provided additional insights into motivations, barriers and influential persons as well as the readiness of PW to adopt recommended practices.

During implementation, we used different data collection strategies based on programme needs and available resources. In Bangladesh, we conducted rapid household assessments over a 6‐month period and service record reviews in selected areas on a limited number of topics to track coverage and practices associated with new processes; this involved using two‐ to three‐page checklists to complement routine monitoring to determine if certain modifications made during implementation were working. In Burkina Faso, we conducted two rounds of exit interviews with ANC users, and in Ethiopia, we carried out a mid‐line service provision assessment including observations and exit interviews. In India, we conducted several studies such as the cost of diets to determine the feasibility of dietary recommendations (Kachwaha et al., 2020) and progress in intervention coverage (Kachwaha et al., 2021). The types of data and information sources are listed in Table 1.

**Table 1:** Steps and data sources to adapt maternal nutrition interventions for integration into ANC services in Bangladesh, Burkina Faso, Ethiopia and India

Steps and data sources	1. Situational analysis and formative research	2. Design and testing of MNI processes and tools	3. Micronutrient supply assessments	4. Household and health provider surveys	5. Monitoring, intensified supervision and special assessments/studies
Bangladesh					
Timeline	October 2014–March 2015	April–May 2015	March–May 2015	June–August 2015	August 2015–October 2016
Data and information sources	– DHS surveys (NIPORT and ICF, 2020) and MNCH programme reports – Qualitative research (Schuler, 2015) on community perceptions and drivers of behaviours, foods accessibility and affordability, potential role of husbands	– Registers and training pilot tested (BRAC, 2015a, 2015b) – Counselling tools, ANC job aids and IEC materials pretested with PW, family members and community members (Alive &Thrive, 2016)	– Desk review and field staff discussions on IFA and calcium supply chain – Review, discussions and alignment of NGO with national protocols	– Survey of RDW (*n* = 2000), PW (*n* = 600) – ANC provider and health volunteers (*n* = 300) – Survey of husbands of RDW (*n* = 1400) (Nguyen et al., 2015, Nguyen, Sanghvi, et al., 2017)	– Routine monitoring by providers, for example, empty micronutrient strips in home visits (BRAC, 2015b) – Data recorded in Mother‐Baby booklet – Rapid surveys for coverage trends and targeting of lagging geographic areas – Content of household visits and trends in practices assessed quarterly
Main findings	– Late ANC visits reduced the number of micronutrient supplements received and counselling opportunities – Cost of IFA and calcium a barrier – Multiple local food sources available but not utilized – Husbands willing to support PW, lack knowledge, for example, benefits for mother and child	– Community agent and provider tasks unclear, for example, for record‐keeping, registration of new pregnancies and referral – Scales difficult to carry during home visits – Forum timing to suit husbands' work, male staff facilitate	– Supplies estimated based on 180 tablets of IFA and calcium – No charge for micronutrients would raise supply projections – Higher budget for supply needs, no cost recovery	– Women consumed 94 IFA and 82 calcium; half had adequate diet diversity – ANC started late, gaps in counselling, micronutrient adherence was not addressed – Food groups available; food insecurity = 10%–11% – Large gaps in provider and women's knowledge	– Staffing, IEC materials, power supply issues for husbands'/community forums – Supervision checklists inadequate for the quality of ANC services – Content of refresher training not targeted to key gaps; too infrequent – Frontline worker skills inadequate, for example, use of registers, mobile phones to contact supervisors and facilitation skills
Programme adjustments	– ANC visits increased to 7 by providers, 14 visits by volunteers – Free provision of supplements – Tailored interventions and IEC materials for improving husbands' support	– Volunteers maintain weighing scales at home – Husband's forums assigned to male staff – Training and IEC materials aligned – Only low‐cost seasonal fruits/vegetables were specified for dietary counselling	– Estimated supply needs based on 100% coverage of PW and adherence to protocols for IFA and calcium supplements – Supply monitoring process and tools specified and staff orientated	– Content of job aids, training and supervision focussed on key gaps – Weighing, EIBF and diet counselling skills made a priority focus of training – Underutilized local foods emphasized in tools – Food diversity explained to PW/families	– Incentive criteria clarified – Data quality is validated through triangulating different sources – Data reviews prioritized – Husband's forum targets set, role of volunteers and facilitators specified – Special staff assigned for ANC quality – Refresher training improved, e.g., peer to peer problem solving and supervision checklist emphasized
Burkina Faso					
Timeline	January–June 2019	July–December 2019	September– October 2019	November–December 2019	May 2020–January 2021
Data and information sources	– Situational analysis (PMA2020, 2018) – Formative research (Ky‐Zerbo et al., 2019), IDI with managers, providers, community agents, post‐partum women, community leaders; FG with husbands, women elders; household trials to define feasible actions – Preliminary results of formative research discussed and codesign workshop to develop interventions	– Based on formative research (Ky‐Zerbo et al., 2019) continuing intervention odesign workshops ‐Design of IEC materials – Training plans, materials, job aids developed	– Training and testing of processes and materials – Supply chain assessment for IFA and malaria control drugs in two regions (Zongo et al., 2019)	– Surveys of RDW women (*n* = 1920), PW (*n* = 800 – Survey of ANC providers (*n* = 80) – Survey of husbands of RDW (*n* = 991) (Kim, Ouédraogo, et al., 2020)	– Joint health team site visits using a checklist – Routine record reviews of coverage and quality – Two rounds of external exit interviews with PW, initially at all health centres and repeated for low performing centres (Sosthène, 2020)
Main findings	– Maternal nutrition policies/protocols not specific; calcium supplements in ANC not approved – Gaps in supervision, monitoring, counselling tools – Local foods are available but PW are not aware – Misperceptions about IFA – Weight measured but not used; lack of tools (e.g., chart), skills	– Providers lacked skills in using tools, inability to prioritize key behaviours – Weak monitoring skills and use of data – Need for clarity on the roles of ANC providers, health centre chiefs and community agents – Need for refresher training and coaching	– Supplies were available but stockouts existed – Interviews with ANC providers did not identify bottlenecks in supplies – More useful information was obtained from direct ANC observations and PW interviews	– On average, women consumed 107 IFA tablets – Only 27% of PW had adequate diet diversity – Delayed start, early stoppage of IFA intake – Lack of knowledge of IFA – Low quality/no counselling reported by PW on diet, IFA, weight gain and breastfeeding in ANC	– Delays in ANC identification/registration – ANC is not provided daily in all centres – IFA stockouts due to poor stock management and high transportation costs from district warehouses to facilities – Poor counselling skills, providers unable to cover all topics in a single visit, lack of skill in tailoring key messages for each PW – Data reviews not used adequately – Community agents' home visits and husbands' engagement inadequate
Programme changes	– Focussed messages for PW based on ‘small do‐able actions' – Potential roles of ANC providers and community agents defined; gaps in counselling addressed through job aids and supervision – Weight gain chart added in health booklets – Assistance of NGO for mentoring of government staff	– Tasks specified for ANC providers, for example, for each trimester of pregnancy – Messages and images improved in job aids and reminder materials for providers – Specific local foods named and illustrated for dietary diversity	– Routine weekly surveillance of stocks and centre level tracking – Messages on 180 IFA tablets per PW accompanied by counselling on adherence – Exit interview surveys were added to validate routine service records	– New job aids added and streamlined materials for community agents – Specific messages and materials for grandmothers and community leaders – Counselling skills strengthened to address barriers and clarify recommended practices	– Counselling focussed on key PW concerns – Daily ANC established at health centres with staff adjustments, for example, sharing staff – Weekly IFA stock surveillance to address gaps; transport costs reduced – MNIs added to data reviews with community agents at facilities; communication of facility chiefs with district management to include MNIs – Data were used to identify lagging centres where community agents were re‐trained
Ethiopia					
Timeline	July–January 2019	February–June 2019	January–March 2019	November–December 2019	December 2019–June 2021
Data and information sources	– Situation analysis (EPHI & ICF, 2019; Hirvonen & Wolle, 2019) – Compilation of existing formative research (Clemmons & Griffiths, 2016) – Dialogue with two regional governments and national staff	– Codesign workshops to contextualize the programme for two regions – Pretesting tools and materials, pilot tests	–h IFA supplement supply system reviewed at facilities, regional hubs and teams at the national level	– Surveys of RDW women (*n* = 344), PW (*n* = 175 – Survey of ANC providers (*n* = 120) (Kim, Sununtnasuk, et al., 2020)	– Routine review meetings, supportive supervision with a checklist for observations and PW interviews – Midline assessment, PW exit interviews ANC observations, record reviews (Alive & Thrive, 2021)
Main findings	– Late start of ANC – Low consumption of IFA and low dietary diversity; use of calcium supplements in ANC not approved – Low community awareness of nutritional needs in pregnancy – Social norms on late ANC, low IFA adherence and knowledge of need and side effects – Diet diversity is influenced by real or perceived affordability, lack of awareness of using seasonal foods and gender bias	– Health workers and managers reported low ANC attendance – Government prioritized maternal health but gaps in nutrition content – Community leaders motivated to raise awareness – Tasks identified for different actors – Codesigning worked well, but few participants from high need areas	– IFA supply requisitions from health centres were not filled due to usage – IFA is not a priority on drugs lists – IFA is not recorded in registers – PW were not counselled on IFA protocols – ANC providers unsure about protocols – Tablets handed out in paper – No IFA counselling	– Regional differences, for example, only 65% of women in Somali and 93% in SNNP consumed any IFA tablets – Food diversity is also region‐specific – ANC started late – No consolidated PW ANC records to track services from different facilities – PE weighed but counselling not given on weight gain – IFA protocol and side effects not explained	– Supportive supervision and record reviews facilitated supplies – Training, record‐keeping initiated – Gaps remained in counselling and husbands'/family's engagement – Need for refresher training and intensified supervision following COVID‐19 – Government mandated reduction in services during COVID‐19, reduced ANC visits by PW – Field observations showed few supervision visits in SNNP – Somali lacked IFA and IEC supplies; families experienced food insecurity
Programme changes	– Options explored for improving early registration, more visits – Potential strategies to reach families for supporting dietary diversity are discussed at the community level – Training and IEC materials developed for IFA and dietary diversity adherence	– Contact points specified and tools developed for health facilities, communities – Nutrition service delivery is given more focus at PHC, health posts, home visits, group meetings – Regional NGOs hired for mentoring and capacity building in SNNP and Somali	– New estimates of IFA supply needs and a 6‐monthly delivery are recommended from regional hubs to district and primary health centre stores during scale‐up – Record keeping for IFA revised at facilities	– Intensified focus on counselling using a few simple messages – Improved tracking of IFA supplies at the point of service and regional distribution hubs – Weight gain calculation and record‐keeping skills – Supervision checklists and record reviews increased	– Advocacy was conducted with district managers to intensify field supervision, including ANC observations, PW interviews and record reviews – Materials and tools streamlined based on observed use; a tracking booklet for PW given extra focus – Enlarged job aids used as posters reminded ANC providers about key tasks – Regular reviews of ANC register data on nutrition
India					
Timeline	January–August 2017	March–June 2018	Mid‐2018	September–December 2017	January 2018–January 2020
Data and information sources	– Information on national policies and state ANC services (Nguyen, Avula, et al., 2021) – Formative research on dietary patterns, food availability, taboos and influences, channels of communication and IFA sources (CMS, 2015)	– Pilot test of community and system processes – Options for husband's engagement tested – Monitoring tools and IEC materials pretested	– Desk review of IFA and calcium specifications, national policies and state procurement rules – Supply chain study on IFA/calcium, tools and skills	– Surveys of RDW (*n* = 1838) andPW (*n* = 667) – Frontline worker surveys (*n* = 520) – Husbands surveys (*n* = 452) (Nguyen et al., 2018; Nguyen et al., 2019)	– Facility and community capacity studies identified gaps; PW coverage issues and the cost of recommended foods defined (Bellows et al., 2020; Kachwaha et al., 2020). – ANC worker knowledge and practices and data use by government staff (Young et al., 2021) – Supportive supervision and routine records
Main findings	– Multiple contact points for PW at facility/community levels – IFA supply gaps; Ca not started – Poor knowledge of PW on micronutrients, diet diversity – Low‐cost foods and family support are enablers of dietary diversity – Limited media but CHW are key	– Gaps in training, mentoring and tools – Job aids needed with specific messages for different actors – Options needed to engage husbands and community	– Detailed diagnostics and planning are required for addressing supply gaps – Weaknesses in existing tools, templates – ANC providers and pharmacists lack skills in assuring stocks	– Gaps in nutrition knowledge and practice in PW and frontline workers – Counselling not done – Supplements available but protocols not followed – Weight gain and dietary diversity are not understood	– Interventions and processes feasible – ANC providers and community workers improved skills/capacity to deliver MNIs – PW improved knowledge and reported better practices; husbands' participation improved but low coverage – Government staff did not use data to make programme adjustments
Programme changes	– Detailed tasks and tools for each ANC contact developed – Materials to fill gaps – Agreement to implement calcium supplementation – Supervision and monitoring – Data review options identified	– Joint supportive supervision with government staff – Data reviews based on service records – Dashboards for MOH – NGO to provide ongoing mentoring and coaching	– Supply system plan – Tools, templates and coaching on supply systems for the government system	– Focus on strengthening supportive supervision – Use of routine data by government staff to jointly identify gaps and solutions – Capacity of ANC providers on weight tracking, dietary counselling skills	– Ministry of health adopted MNI protocols in routine ANC nationally – Ongoing refresher training and supervision feedback on all levels – Adoption of tools and interventions at the state level to institutionalize MNIs in ANC – New community platforms engaged for greater male participation

Abbreviations: ANC, antenatal care; BRAC, national NGO; Ca, calcium; FG, focus groups; ICDS, Integrated Child Development Services; IDI, in‐depth interviews; IEC, information, education and communication; IFA, iron folic acid; MNCH, maternal newborn and child health; MNI, maternal nutrition interventions; MOH, Ministry of Health; NGO, nongovernmental organization; PW, pregnant women; RDW, recently delivered women; SNNP, Southern Nations, Nationalities and Peoples region in Ethiopia; UP, Uttar Pradesh.

We identified gaps and opportunities through different sources of data and information and used the findings to develop locally relevant interventions. During implementation, bottlenecks that were not anticipated in the design phase were identified and rapidly addressed through supervision and monitoring. When new issues were identified, strategies were continuously adjusted. Each country used existing mechanisms and resources available for tracking progress and detecting needs such as gaps in supplies, lack of counselling, or incorrect use of ANC registers and information, education and communication materials. Frequent meetings with providers and authorities responsible for ANC services were important for triangulating information to arrive at decisions on how to refine the interventions. We refined the programme models throughout implementation with the goal of improving effectiveness, feasibility and scalability of approaches designed to strengthen the quality of nutrition services and adoption of nutrition practices. Four country‐specific models of nutrition integrated into ANC were finally developed.

### 2.1 Ethical considerations

Ethical approval for all research studies was obtained from the Institutional Review Boards of the designated countries and from the International Food Policy Research Institute.

## 3 RESULTS

The main steps applied for adaptation and integration and data sources for developing the MNIs in each country are presented in Table 1. The final MNI protocols that resulted from this process are shown in Table 2. For each MNI, we summarized the lessons learned for operationalizing these protocols in the context of existing ANC programmes (Table 3). The integrated models reflect global recommendations, national policies and country‐specific patterns of ANC service delivery such as ANC locations, types and frequency of services, task allocations across different cadres of workers, and supply and information systems. In this section, we describe the adapted MNIs, supports to services needed to deliver the MNIs at health systems and community levels and key characteristics of each country's programme model.

**Table 2:** Protocols for four maternal nutrition interventions integrated into ANC in Bangladesh, Burkina Faso, Ethiopia and India

Interventions	Bangladesh	Burkina Faso	Ethiopia	India
1. Micronutrient supplementation	IFA and calcium are included; 180 doses per PW; supply chain protocols specified; distribution is free of cost from facilities, also permitted during home visits by ANC providers; counselling and record‐keeping are well‐defined; volunteers but not health workers, receive cash incentives for PW completing protocols; IFA, calcium included in initial and refresher training; and in monitoring and supervision tools	IFA is included in ANC, but not calcium; dosage of one tablet daily from the first ANC contact until 42 days post‐partum; there is an established supply chain. Stock monitoring is conducted weekly using WhatsApp; distribution of IFA to PW by ANC providers is free of cost and from facilities only; counselling messages are well‐defined; the number distributed per PW is recorded and included in the supervision checklist	IFA included in ANC, but not calcium; dose set at 90 IFA per PW; distribution is by ANC providers permitted from facilities (health centres and health posts) only; supply chain issues recently addressed for forecasting, requisitions, distribution and record‐keeping; stock replenishment plans improved; IFA related counselling messages and record‐keeping are specified	IFA and calcium are included in ANC; doses are 180 IFA, 360 calcium tablets per PW; distribution sites include facilities, community‐based outreach sessions and home visits for selected hard‐to‐reach households; supply chain improved, staff trained on protocols; training provided to pharmacy in‐charges and health managers on forecasting, procurement, distribution and monitoring; IFA and calcium counselling messages are defined
2. Weight gain monitoring	Tasks for weighing are specified; staff are trained to calculate weight gain; weighing sites include community level with support from community health volunteers; for tracking weight gain, home‐based posters and ANC registers are used with spaces for dates and weights	Steps are specified for calculation of weight gain and counselling; weighing sites are facilities only; for tracking weight gain, PW's Health Booklet (this has a weight gain chart) and ANC registers are used with spaces for dates and weights; intervention is included in supervision checklist	Protocols for weight gain calculation specified; steps for weighing displayed at ANC/weighing sites; messages based on tracking weight gain defined; weight gain is integrated into training and supervision tools; ANC registers and PWs nutrition card	ANC providers are trained and supervised to monitor weight gain at facilities and community‐based monthly outreach sessions for ANC; counselling messages are defined; intervention is included in job aids, training tools and supervision checklists; weights and dates are entered in ANC registers; weight gain monitoring included in home‐held IEC materials
3. Dietary counselling	Locally available seasonal foods are clearly illustrated, and amounts and number of meals for each trimester; ANC providers conduct one food demonstration for families at the first home visit; training, supervision and record‐keeping include counselling dates; same messages are included in community events	Specific affordable local foods are selected based on seasonal availability in each region; amounts are illustrated in job aids; messages and images are included in training tools for facility and community agents; intervention is included in the supervision checklist and space has been added in the ANC register for counselling topics for each PW	Acceptable and available local foods tailored to seasonal availability per region; amounts and meals specified; illustrated job aids and posters used at ANC locations; dietary counselling is integrated into training and supervision tools; recording of dates of dietary counselling is specified in ANC guidelines	Acceptable local foods and the amounts number of meals are illustrated in counselling job aids; messages and images are added in training tools and the supervision checklist; for monitoring, recording of dates of counselling has been specified. Messages and images have been added to community mobilization tools and in materials for husband's forums
4. Breastfeeding counselling	Messages specified in job aids for EIBF and EBF; training, supervision aligned; counselling dates in the ANC registers	Messages specified in job aids and dialogues with grandmothers on breastfeeding, skin to skin contact; training, supervision tools aligned; space added for topics in registers	Job aids specify EIBF and EBF; training and supervision aligned; counselling dates in ANC registers	EIBF and EBF specified for counselling PW; training and supervision tools aligned; counselling dates added in ANC registers

Abbreviations: ANC, antenatal care; BRAC, national NGO; EBF, exclusive breastfeeding; EIBF, early initiation of breastfeeding; ICDS, integrated child development services; IFA, iron and folic acid; MNCH, maternal newborn and child health; PW, pregnant women.

**Table 3:** Support services for delivering MNIs integrated into ANC services in Bangladesh, Burkina Faso, Ethiopia and India

	Bangladesh	Burkina Faso	Ethiopia	India
Country partners	Ministry of health's ANC and Nutrition units provided policies and oversight; BRAC (NGO) MNCH regional and subdistrict managers directed outreach and community services. A&T staff helped to develop the MNIs and provided technical assistance	Ministry of health's ANC and Nutrition units provided policies; service delivery at facilities and community level directed by district health teams; national NGO coached government staff. A&T staff helped to develop the MNIs and provided technical assistance	Ministry of health's ANC and Nutrition units provided policies; service delivery at facilities and community level under regional and district health teams; NGOs coached government staff. A&T staff helped to develop the MNIs and provided technical assistance	Ministry of health's National Health Mission provided policies and service delivery; Ministry of Women and Child Development, ICDS provided support; NGO coached government staff. A&T staff helped to develop the MNIs and provided technical assistance
Support services to deliver MNIs				
Facilitating early ANC registration and timely ANC contacts	– ANC providers assigned monthly targets for new ANC registrations based on the catchment area population – Structured schedules and task allocation given to ANC providers and community volunteers to cover 100% PW through home visits – Intensified monitoring of ANC coverage and additional supervision – Cash incentive for volunteers to include early ANC	– Community agents trained to encourage women to enrol early and complete planned visits, obtain IFA and monitor their weight gain at ANC sessions – Community leaders are sensitized to help mobilize families through community meetings with family members to register PW for ANC and increase the number of ANC contacts at health facilities	– Pregnancies are identified through home visits by community volunteers and health extension workers at the health post level; newly identified PW are asked to attend their first ANC visit at primary health care centres – Enhanced services, counselling and supplements are provided at health posts, located close to communities – Dialogue with family members during home visits to encourage ANC contacts	– Monthly outreach sessions are conducted in communities for ANC (village health and nutrition days) to improve convenience for PW – Early and frequent ANC promoted in home visits by community staff – Community leaders convince reluctant families to send PW to attend ANC – Special campaigns to drive up attendance at ANC – Community‐based programmes (e.g., ICDS) and workers engaged to promote early and multiple ANC
Building the capacity of managers and ANC providers	– MN content added to existing operations of NGO (BRAC) MNCH programme – Video on delivering maternal nutrition used to standardize quality during decentralized cascade training used for scale‐up – Field‐based practical sessions used with a focus on problem‐solving to strengthen ANC provider and volunteer skills – Monthly feedback from supervision and problem‐solving; training and follow up on monitoring indicators and use of tools and data	– Pool of trainers for ANC providers; also conduct supervision after training – Orientation on MN service provision for district health teams, pharmacists, facility heads, maternal health staff – Initial/refresher training for ANC providers, community agents – Supportive supervision review meetings for skills development – 3‐monthly reviews of MNI monitoring data focussed on identifying and addressing gaps – NGO technical support and coaching for government staff	– Skills‐based training and practice sessions to prepare regional MOH master trainers – Orientation sessions for district and facility‐based managers; quarterly updates to managers for problem‐solving – Two‐day skills‐based training for ANC service providers and monitoring staff; 1‐day session for community volunteers and village heads; 1‐day refresher training after the shutdown – NGO technical support and coaching in Somali, SNNP	– Advocacy to fill ANC provider vacancies; additional support from ICDS workers through strengthened coordination processes to expand capacity and share tasks across sectors – Multi‐layered, cascade training on MN services – Microplanning jointly with government staff to reduce gaps in equipment and supplies – Dashboards and review meetings for routine data and supervision – Mentoring approach used during joint field visits – Coordination of health and ICDS – NGO technical support and coaching for government
Engaging community and family members to support adherence to recommended practices	– Tailored protocols, tools for husbands' forums facilitated by male staff and messages given on household budgets and benefits for the child – Tracking and follow up on participation of PW husbands; targets set for two forums per husband, preferably in the second and thirrd trimesters – Cash for transportation and in‐kind incentives such as seeds to compensate for lost wages and travel costs	– ‐Community agents motivate husband's attendance at ANC – Husbands oriented on IFA and food access in home visits, and group meetings – PW is encouraged to ask for support from husbands – Family‐based illustrated tools for community agents to use during dialogues – Community leaders sensitized to mobilize community members and family members – Family counselled on support	– Community opinion leaders and networks engaged in supporting MN in SNNP, Somali – Community group meetings (called ‘PW conferences') to encourage women to engage husbands in meeting needs for food and micronutrient supplies – Home visits conducted by health extension workers and community volunteers; include dialogue with husbands and family members	– Husbands' engagement encouraged through village committees and local government leaders – Special events for engaging husbands in communities – Community workers trained to contact male family members during home visits – Materials tailored for messages on the role of husbands, types of support husbands can provide and benefits for the unborn child
Improving PW's knowledge, beliefs, self‐efficacy	– Messaging on self‐efficacy, how to avoid/manage IFA side effects and extra food groups – Food access is addressed by an emphasis on seasonally available affordable foods – Husbands informed about benefits for the child; family budgets and cost of seasonal, affordable foods discussed – Gender bias addressed in social mobilization events, through videos and in husbands' forums	– Improved counselling by ANC providers and engagement of family members by community agents – Families engaged during home visits and community meetings to support PW – Community agents trained repeatedly on these issues – Messages on food varieties are designed to be feasible, with emphasis on local, seasonal foods to build self‐efficacy and access to food	– ANC providers trained to understand individual barriers and mentor/coach for building PW skills and self‐efficacy – A reminder tool (PW tracking chart) is used to facilitate discussion on barriers; current practices and negotiated future practices recorded and discussed at ANC visits – Local seasonal and affordable foods identified for dietary diversity in Somali and SNNP regions	– Focus on improving counselling to address knowledge and self‐efficacy in PW and tailor counselling to the PW family's socioeconomic situation – Content of dietary counselling on affordable, locally available foods – Gender bias is addressed in community meetings and special forums to highlight the risks of undernourished PW for child/PW – Elders, husbands, mothers and mothers‐in‐laws were asked to attend community events and home visits

Abbreviations: ANC, antenatal care; BRAC, national NGO; ICDS, integrated child development services; IFA, iron and folic acid; MN, maternal nutrition; MNI, maternal nutrition interventions; MNCH, maternal newborn and child health; PW, pregnant women; SNNP, Southern Nations, Nationalities and People's region in Ethiopia.

### 3.1 Content of MNIs

The four MNIs existed in principle at the national policy level in all the countries with a few gaps (e.g., calcium supplementation was not included in ANC in Burkina Faso and Ethiopia). However, ANC in all countries had missing elements in MNI protocols and did not adequately specify nutrition‐related tasks for facility‐based and community‐level service delivery, supervision and monitoring (Sanghvi et al., 2022). Although IFA supplementation was better addressed than the other nutrition interventions, knowledge gaps related to all MNIs were observed among ANC providers and PW. The adherence of PW to recommended nutrition practices was low (Kim, Ouédraogo, et al., 2020; Kim, Sununtnasuk et al., 2020; Nguyen et al., 2015, 2018). Inadequate understanding of MNIs and poor skills in counselling among providers were common (Sanghvi et al., 2022). We developed tools for strengthening counselling quality with specific content for each MNI based on global recommendations. The tools provided contextualized content and facilitated an interactive approach using questions and answers. Counselling was encouraged to be conducted at different sites by all levels of health workers including at facilities, outreach sites, group sessions and home visits.

#### 3.1.1 Micronutrient supplementation

IFA was the common micronutrient supplement provided during ANC in the four countries. National IFA protocols in Bangladesh, Burkina Faso and India adopted one daily tablet to be consumed for 6 months of pregnancy (a total of 180 IFA tablets), as globally recommended. Ethiopia recommended ‘at least 90 IFA tablets', which was the cutoff used as a coverage indicator in national surveys. Supplements were distributed free of cost in all ANC services. In Bangladesh and India, calcium supplementation was also included in the ANC guidelines (Nguyen et al., 2015, 2018). In Uttar Pradesh, India, a protocol for calcium supplementation existed, but frontline workers were not trained or asked to distribute or counsel PW about them, and supplies were not available. Routine calcium supplementation during pregnancy was not yet approved in Burkina Faso and Ethiopia, pending further evidence on calcium deficiency.

Enhanced counselling on micronutrient supplementation and recording and tracking of the dates and numbers of doses distributed were adopted and strengthened in all the country models. A&T programmes addressed the ‘what', ‘why', ‘when' and ‘how' to improve adherence to IFA or calcium supplementation by strengthening counselling content. For ‘what', the action was explained (e.g., taking a daily IFA or calcium supplement for 180 days); for ‘why', we presented the benefit of taking IFA or risk of not taking them such as anaemia, post‐partum haemorrhage and poor child cognition; for ‘when', we specified timing for taking IFA (e.g., after a meal preferably at night before sleeping); for ‘how', we explained that IFA should be taken with plenty of water, fruits and vegetables to prevent constipation and enhance absorption. We also encouraged family members to remind and support PW to complete the full regimen. Micronutrient supplement distribution during home visits by nonmedical, trained and supervised community workers was permitted in Bangladesh and India to improve coverage. In Burkina Faso and Ethiopia, supplements were distributed by ANC providers only. A&T programmes also promoted early initiation of and multiple ANC contacts, helping PW to obtain sufficient micronutrient doses throughout their pregnancy.

#### 3.1.2 Weight gain monitoring

Although weighing was a routine procedure during ANC visits, calculation of weight gain, compared with recommended weight gain and counselling on healthy weight gain were not well implemented in any location (Kim, Ouédraogo, et al., 2020; Kim, Sununtnasuk, et al., 2020; Nguyen, Sanghvi, et al., 2017; Nguyen et al., 2019). Competencies of service providers in taking accurate measurements, calculating weight gain, record keeping, comparing weight gained with recommended ranges and advising about adequate weight gain remained a challenge even after training. Tools were developed such as the ‘Maternal Nutrition Follow‐up Card' in Ethiopia for recording the weights and weight gain of individual PW. A family‐based poster with space to record weights was used in Bangladesh and weight gain charts were incorporated into the women's health booklet in Burkina Faso. In India, better use of the existing maternal and child cards for recording weights was reinforced and a family‐based poster to engage family members in supporting frequent weight gain monitoring of PW was developed.

#### 3.1.3 Dietary counselling

Common gaps in counselling were giving general messages such as ‘eat more food varieties' without specifying local food examples, providing names of too many foods (e.g., in all 10 food groups), thereby overwhelming PW, not reinforcing positive elements including several food groups already being consumed, not adjusting messages for seasonally available foods and costs, not having a dialogue through questions and answers, not addressing PW concerns and not engaging family members (especially husbands and mothers‐in‐law). The integrated country models (developed by A&T and government authorities) specified protocols for counselling on dietary diversity and amounts of foods to be consumed over the course of pregnancy, with increasing amounts and frequencies of meals and nutritious snacks in the second and third trimesters of pregnancy. Other counselling messages included the importance of consuming one food item daily from each of the five specified food groups, examples of local seasonally available foods andthe importance of adequate meals (and snacks) and food amounts to maintain a healthy range of weight gain. Illustrated job aids in all country programmes provided local examples of affordable, accessible, seasonally available and nutrient‐rich foods. Burkina Faso, Ethiopia and India invested in multiple rounds of training, orientation sessions and refreshers on dietary counselling, with a focus on individually tailored messages and problem‐solving. Counselling was monitored through supervision, service record reviews and assessments (e.g., exit interviews). All country models addressed the participation of family members, particularly husbands in discussions to enable women's access to diverse foods. To improve family support, husbands' forums were developed in Bangladesh and India and community workers engaged family members during home visits in Burkina Faso, Ethiopia and India. In Burkina Faso, husbands were also reached through group meetings where men usually gather.

Record keeping for tracking the provision of dietary counselling was a missing element in all locations and was added to ANC monitoring and supervision processes. For example, we added columns in the ANC registers for recording dates and contents of counselling. We also designed new supervision checklists and PW's booklet to record dates, locations and contents of ANC visits. These helped to track the number of visits and counselling provided so that mid‐course corrections could be made through refresher training, revised job aids or additional supervision visits.

#### 3.1.4 Breastfeeding counselling

Common weaknesses in counselling were not prioritizing the topic of breastfeeding during ANC, not demonstrating skills of positioning and attachment, giving messages instead of engaging in dialogue through questions and answers, not listening to the concerns of PW and family members, not discussing anticipated problems (e.g., return to work), not tailoring the solutions to the PW's situation and not clearly communicating the risks associated with not practicing EIBF and EBF. Protocols for counselling during ANC visits were revised to address specific concerns and gaps in PW's breastfeeding knowledge. Counselling guidelines for breastfeeding included the importance of early initiation within 1 h and 6 months of EBF, how to place the newborn on the chest, preventing and managing common difficulties and manual expression of breast milk. Messages and support for EIBF and EBF were integrated into ANC job aids, training, intensified supervision, monitoring and family engagement tools. Anticipatory guidance on arranging for timely initiation immediately after delivery, disallowing prelacteal feeding and keeping mothers close to their infants in the first 6 months were added to the messages that were already being given on breastfeeding in most ANC programmes. ANC provider skills were reinforced through training, tools and monitoring to facilitate routine implementation at facility‐based and community contacts with PW and family members.

### 3.2 Supports and inputs to strengthen service delivery

To enable the delivery of effective MNIs integrated into ANC, we needed to address underlying barriers such as delayed and infrequent ANC visits, low capacity of health personnel in maternal nutrition service delivery, weak linkages with families of PW and lack of emphasis on strengthening PW's self‐efficacy. Steps taken to address these barriers included facilitating early initiation of and more frequent ANC contacts, building the nutrition capacity of managers and ANC providers to deliver MNIs, strengthening family and community engagement to support PW and building the knowledge and self‐confidence of PW to adopt the recommended nutrition practices (Table 3). The country models reflected differences in the structure of ANC services, strengths and maturity of ANC programmes, types and performance levels of service providers, patterns of ANC utilization and the influence of household and community factors. In Burkina Faso and Ethiopia, only health providers deliver ANC services, whereas in Bangladesh and India, community health workers are actively involved in counselling PW, distribution of micronutrient supplements during home visits and family engagement for support during pregnancy. Bangladesh and Burkina Faso have well‐functioning supply systems and provider capacity, but India and Ethiopia have weaker health systems for nutrition service delivery during ANC, requiring more systems strengthening efforts. Task allocation among cadres of ANC providers and their performance levels differed across the countries. In Burkina Faso, coverage of facility‐based ANC visits was high and paid community health agents conducted home visits and family and community engagement activities. Bangladesh also achieved a high coverage of ANC contacts through home visits, conducted by salaried ANC providers with incentivized health volunteers who shared some tasks such as counselling. Ethiopia's health extension worker cadre was the predominant ANC provider and conducted facility‐based services as well as family and community engagement activities. In India, Bangladesh and Ethiopia, strong linkages between health workers and households and communities were in place. Examples of lessons learned about health systems strengthening and community engagement strategies are summarized in the Supporting Information Table S1.

### 3.3 Country programme models developed by A&T

*The Bangladesh model* was based on a nationally scaled‐up maternal nutrition and child health programme that was being implemented by an NGO (BRAC) to extend essential services to communities. BRAC's integrated ANC programme used a performance improvement cycle to build service provision capacity through task allocation, ongoing training, job aids, supervision and monitoring feedback, and performance‐based cash incentives for community volunteers. Husbands' forums were structured so that each husband would attend the forums at least twice during their wife's pregnancy. The ANC service platform with high coverage and frequent contacts with PW was strengthened further with additional nutrition service content, which significantly improved IFA and calcium adherence, weight gain tracking, dietary diversity and EBF practices (Nguyen, Sanghvi, et al., 2017).

*The Burkina Faso model* centred on improving ANC services delivered through government primary health care centres and strengthened linkages to community health agents. The government's district management team and health centre chiefs were involved in supporting and prioritizing MNIs within the package of ANC services. Despite high coverage of facility‐based ANC visits, the frequency of ANC visits was low before integrating MNIs (Kim, Ouédraogo, et al., 2020; Kim, Rock, et al., 2021). With additional training and support to build government health system capacity by integrating MNIs into ANC services at the facility and community levels, the model led to significant improvements in early initiation of and frequency of ANC contacts, IFA adherence and early breastfeeding practices (Kim, Rock, et al., 2021).

*The Ethiopia model* was tailored to two distinctly different regions—Somali (pastoralist region) and SNNP (Southern Nations, Nationalities and Peoples, agrarian region). Both regional versions of the country programme models focussed on improving MNIs integrated into ANC services in primary health care centres and local health posts of the government health system. Capacity building of health centre staff and health extension workers for service delivery to PW was a priority in both regions; existing community activities such as group education and home visits were used as channels for reinforcing key MNI messages. Training and orientation sessions and ongoing coaching to government staff for continued adaptation of the service provision and community activities by partner NGOs familiar with each region led to improved coverage of MNIs, dietary diversity and IFA adherence (Kim, Sununtnasuk, et al., 2021).

*The India model* was developed and implemented in Uttar Pradesh state through a state‐run government health system with a relatively weak ANC platform. The model combined facility and community‐based activities tailored for two existing government programmes (Health Department and Integrated Child Development Services, ICDS). The model strengthened MNI counselling during home visits conducted by ICDS workers. With a low frequency of contacts and poor nutrition content in ANC services and several system challenges (Nguyen et al., 2019), a longer time was required to strengthen the ANC programme and show progress (Kachwaha et al., 2021). However, the programme managed to improve coverage of MNIs; women in the integrated nutrition and ANC areas were significantly more likely to have received counselling on core nutrition messages than those in the comparison areas (64% vs. 55% for dietary diversity, 69% vs. 51% for adequate meal intake, 50% vs. 40% for weight gain, 89% vs. 81% for IFA, 68% vs. 55% for calcium and 61% vs. 54% for breastfeeding). Dietary diversity, IFA consumption and EBF were modestly improved (Nguyen, Kachwaha, et al., 2021).

## 4 DISCUSSION

In our paper, we provide examples of *how* four country models were developed to integrate and enhance MNIs into ANC services. We describe lessons learned from developing the models through a systematic process to contextualize a package of nutrition interventions. Our findings demonstrate the importance of combined facility‐ and community‐based approaches to improve service provision and adoption of recommended maternal nutrition practices.

Our findings were similar to experiences from other initiatives for incorporating global recommendations within national health services. Designated periods of field implementation and adjustment were necessary to increase the relevance and acceptability of interventions in large‐scale programmes (Fischer et al., 2016). The Integrated Management of Childhood Illnesses initiative (Lambrechts et al., 1999) and country applications of WHO's ANC guidelines (Barreix et al., 2020) were guided by country priorities and national regulations and policies and were then thoroughly tested for the feasibility of service delivery through the health system. Our approach was also similar to that of the Saving Newborn Lives initiative (Tinker et al., 2010) for adapting maternal and newborn health services, where the steps involved incorporating global recommendations into programme protocols, building system readiness to implement services through well‐defined tasks and staff skills development, provision of training tools and improving equipment/supply chain specifications and record‐keeping protocols. Similar findings have been reported on the importance of clear and comprehensive guidelines and protocols, a skilled workforce, availability of equipment and supplies and adequate resources to meet needs (De Brouwere et al., 2010).

Before programme design, pre‐existing gaps and challenges in service provision protocols, micronutrient supply chains, monitoring and supervision, interpersonal communication and weak community linkage were identified (Sanghvi et al., 2022). Despite different contexts, there were some common actions to strengthen MNIs within ANC across the countries, including (a) specific health systems strengthening actions are required for integrating nutrition interventions (e.g., improving access to ANC through community outreach activities and task‐shifting to trained and supervised community workers); (b) reducing underlying barriers to improving provider and community worker skills and motivations and uptake of interventions by PW are critical; and (c) data from diverse sources should be used strategically and continuously before and during implementation to inform prioritization and decision‐making.

Our programme models benefited from responsive feedback through frequent supervision and monitoring accompanied by data review meetings with implementers and follow‐up on actions to remedy problems identified. With each new information obtained during adaptation and implementation, new issues were identified and strategies were continuously adjusted. Some of the innovations and programme adjustments included focusing heavily on information from health managers/providers/community members in Burkina Faso and Ethiopia at the start to address anticipated operational barriers; developing tools for self‐tracking of ANC visits by PW in Ethiopia; overcoming logistical barriers by sharing transportation costs for IFA supply distribution across areas; standardizing providers' and community workers' training content through videos; using mass media to model supportive social norms (e.g., supportive roles of mothers in law and husbands in Bangladesh and India); engaging religious leaders in Ethiopia and community leaders in India to encourage early initiation of and frequent ANC contacts; and using data strategically to motivate decision‐makers to prioritize MNIs in ANC.

This study contributes to reducing missed opportunities in delivering nutrition interventions in health services (Heidkamp et al., 2020; Shekar et al., 2021) by providing detailed insights on how we integrated MNIs in four large‐scale ANC services. WHO narrowed the gap between maternal nutrition research and health service practices by providing a synthesis of evidence and guidelines (WHO, 2016, 2017). However, the presence of recommendations and programme platforms for service provision does not assure their adoption. Upfront investments were key for learning how to adapt and integrate the MNIs within existing services by understanding the strengths and challenges in ANC provider performance, programme platforms that reach PW and opportunities to leverage community networks to support ANC service use and nutrition practices.

Our experience provides insights into a systematic process for integrating MNIs into ANC in diverse settings. In summary of our numerous lessons learned, we provide the following 10 steps: (1) review current policies and protocols; (2) synthesize findings from formative research and other data analyses to identify key areas of intervention; (3) identify service delivery gaps by assessing existing approaches that relate to the delivery of nutrition interventions in ANC; (4) document the gaps specifically in tasks and staff skills for implementing MNIs; (5) assess the adequacy of current frequency, coverage and quality of training and related tools and job aids; (6) review the need for improved equipment/supply chain and record keeping protocols; (7) document the current use of data to monitor delivery of MNIs and how monitoring data are used to improve the coverage and quality of MNIs; (8) identify the existing community and family linkages to improve ANC service use specifically for MNIs; (9) share the above findings with stakeholders from the health system, academic institutions and the communities; (10) organize codesign workshops to discuss findings, fill in critical gaps in background information, develop feasible processes for improving MNIs in ANC and build joint ownership.

A unique aspect of this paper is the focus on scalability. Working on models for large‐scale programmes required greater attention to system‐wide improvements rather than focusing on health facilities and communities alone. To help transfer our lessons learned to other countries, the theory of change illustrates the barriers commonly faced in LMICs when attempting to strengthen nutrition interventions delivered through ANC on a large scale. We provide examples of lessons learned and innovations developed in our country's programmes to implement the strategies listed in the ToC.

## 5 CONCLUSIONS

The evidence on maternal nutrition's foundational role in determining lifelong and intergenerational health and productivity has been growing (Victora et al., 2021). Nutrition was one of five content areas recommended globally for integration into ANC services (WHO, 2016).

This paper synthesizes immense experiences and lessons learned from working in four different countries to integrate a package of intensified nutrition interventions in existing large‐scale ANC programmes, using a systematic process to contextualize the interventions and reduce gaps between evidence, policies and practice of nutrition service delivery. In addition to strengthening service provision through facilities and outreach, we developed community and family engagement strategies linked to ANC services to generate support for PW and to improve ANC use and adoption of nutrition practices. The experience of integrating maternal nutrition in ANC in diverse contexts can provide operational insights for accelerating progress in addressing missed opportunities for nutrition within health services more broadly.

## AUTHOR CONTRIBUTIONS

Tina Sanghvi, Phuong Hong Nguyen, Manisha Tharaney and Sebanti Ghosh conceptualized the paper. The country models and process of adaptation and integration were documented by Sebanti Ghosh, Zeba Mahmud, Tamirat Walissa, Maurice Zafimanjaka, Robert Karama and Elana Landes Dhuse. Tina Sanghvi developed the theory of change. Phuong Hong Nguyen, Sunny S. Kim and Tina Sanghvi conducted the analyses and interpretation of lessons learned. The manuscript was drafted by Tina Sanghvi and Phuong Hong Nguyen and edited by Jessica Escobar‐Alegria and Sunny S. Kim. All authors read and approved the final submitted manuscript.

## CONFLICT OF INTEREST

The authors declare no conflict of interest.

## Supporting information

Supporting information.

## Data Availability

The data that support the findings of this study are available in the tables/figures and in the Supplementary Information of this article.
