# Supplementary material for: Process of developing models of maternal nutrition interventions integrated into antenatal care services in Bangladesh, Burkina Faso, Ethiopia and India
Source: Matern Child Nutr. 2022 Jun 14;18(4):e13379. doi: 10.1111/mcn.13379 (PMC9480954; doi:10.1111/mcn.13379)
Supplement: Supplementary file 1 — Supporting information. [file MCN-18-e13379-s001.docx]

**Supplemental Table S1. Examples of lessons learned about health systems and community level engagement strategies used for integrating MNIs in ANC services in Bangladesh, Burkina Faso, Ethiopia, and India**

| **Country** | **Health system level** | **Community level** | **Information sources** |
| --- | --- | --- | --- |
| Bangladesh | -Key areas for improvement included the number of ANC contacts, nutrition content and counseling skills of ANC providers, and monitoring and record keeping for four MNIs.  -New tools and repeated training of staff in their use, with frequent follow up from supervisors were used to address gaps.  -Routine monitoring of service delivery specifically designed for the four MNIs, and periodic external validation of routine data through rapid assessments were vital for adjustments operational details for improved implementation. | -Low husbands’ attendance led to community volunteers being engaged to remind husbands about dates and venues of forums.  -Husbands’ forums were conducted by male field managers; tools included hand-outs specifying 10 steps for husbands to support pregnant wives, and a family poster with dates of upcoming forums.  -Husbands’ participation improved through using popular audio tapes with key messages, agricultural extension inputs and reimbursement of transportation costs for attending forums.  -Community video events were used to visually portray husbands and mothers in law supporting maternal dietary diversity and enabling pregnant women to attend ANC.  -Payment required for micronutrient supplements was identified as a barrier and later provided free of cost, as per government policy | Qualitative studies (Schuler, 2015), pilot testing (BRAC, 2015), observations of existing ANC services, and surveys of ANC providers, community volunteers and recently delivered women (Nguyen *et al.*, 2015, Nguyen *et al.*, 2017) . |
| Burkina Faso | -IFA supply gaps due to bottlenecks between health centers and district supplies were addressed  -Mobilizing community agents to address late ANC enrollment and home visits for engaging families needed several cycles of refresher training, meetings with supervisors and new job aids  -Improving poor counseling skills of ANC providers e.g., difficulty in individualizing priority topics among several messages led to identifying a few priority messages and reinforcement during supportive supervision.  -Ongoing quarterly meetings were established between head nurses and health district management teams for progress reviews to maintain a focus on maternal nutrition in the integrated ANC visits.  - Frequent field supervision visits tracked the progress of implementation in the early stages and enabled joint problem-solving with managers and providers. | -Motivating community health agents to expand their work with families of pregnant women was considered key to addressing several issues including early and frequent ANC visits, husbands’ engagement, and adherence to IFA and dietary diversity practices.  -New monthly meetings between head nurses and community agents were established to review progress and reinforce key practices.  -The capacity of community agents was strengthened in using new tools that were specially developed for community influential persons to reinforce maternal dietary diversity, adherence to IFA, breastfeeding and early ANC enrollment. | Qualitative studies (Ky-Zerbo *et al.*, 2019). observations of existing ANC services, and household and provider surveys (Kim *et al.*, 2020a, PMA2020, 2018) were the critical sources of data for shaping the initial design of four MNIs. Supervision visits, routine monitoring data and mid-line assessments (Zongo *et al.*, 2019, Sosthène, 2020) provided data and information for addressing operational gaps during early stages of scaling up |
| Ethiopia | -Supportive supervision addressed priority gaps including, lack of counseling at each ANC contact, poor skills and tools for weight gain monitoring and record keeping, and steps not taken to assure availability of IFA supplies and IEC materials in health centers and health posts.  -Supervisors were asked to facilitate updated record-keeping at health facilities, and to add interviews with ANC clients at each field visit for identifying quality issues.  -Multiple supervision and coaching visits by technical assistance teams during early implementation also collected feedback on persistent gaps.  -Monitoring feedback provided to frontline workers and mid-managers continued building their capacity beyond formal training sessions.  -Reinforcing a few key problem areas and discussing solutions to specific issues directly with ANC providers, repeatedly in every meeting, were effective in reducing service gaps. | -Baseline data showed the need for increasing care-seeking for early registration of PW for ANC.  -Feedback on inadequate husband’s involvement was discussed in consultation with health extension workers and community volunteers.  -In Somali region, the role of religious leaders was explored in engaging husbands for improving access of pregnant women to consume nutrient rich foods for dietary diversity; orientation sessions used for community engagement and tracked through supervision visits. | Baseline data showed where the gaps were and differences in SNNP and Somali regions (Kim *et al.*, 2020b). Existing qualitative research findings were used (Clemmons and Griffiths, 2016). Secondary analysis of existing surveys on food access was used for dietary diversity interventions (Hirvonen and Wolle, 2019). Participatory co-designing workshops used to cross-check feasibility of the proposed program design. The workshops were a part of formative assessments and strategic decision-making e.g., on task allocation and resources during the design of MNIs. |
| India | -Routine data validation and data visualization skills among health staff were strengthened.  -Compiling monitoring data and feedback from supervision visits into ‘report cards’ facilitated discussions during review meetings at subdistrict level and caught the attention of senior staff  -Capacity was strengthened for improving micronutrient supply projections, requisitioning processes, record keeping and monitoring, and managing distribution.  -Monthly IFA and calcium stock and consumption data were collected from district and subdistrict pharmacists and ANC providers and reviewed at meetings to discuss issues.  -Counseling skills and understanding of each pregnant woman’s family constraints by frontline providers were addressed; a few priority messages were used | -Husband’s/men’s attendance at community meetings was a challenge due to employment related migration.  -Male staff facilitated meetings with male members of the community as gender dynamics needed to be addressed; frontline workers are women and discouraged from male interactions.  -To leverage another sector (ICDS program) that had extensive community level presence, coordination with health and ICDS workers was strengthened.  -Orientation was provided to both health and ICDS workers in providing dietary counseling and using the job aids.  -Home visits by both cadres of workers were used to drive up timely and frequent ANC visits. | Existing qualitative research (CMS, 2015), household and provider surveys (Nguyen *et al.*, 2019, Nguyen *et al.*, 2018), supply chain and other system strengthening assessments and data reviews based on routine record-keeping were key information sources required for designing MNIs. Special studies and assessments (Bellows *et al.*, 2020, Young *et al.*, 2021, Kachwaha *et al.*, 2020). |

ANC=antenatal care, IFA=iron and folic acid supplements, MNIs=maternal nutrition interventions, PW=pregnant women, SNNP= Southern Nations, Nationalities and Peoples region in Ethiopia

**References**

Bellows, A. L., Kachwaha, S., Ghosh, S., Kappos, K., Escobar-Alegria, J., Menon, P. & Nguyen, P. H. 2020. Nutrient Adequacy Is Low among Both Self-Declared Lacto-Vegetarian and Non-Vegetarian Pregnant Women in Uttar Pradesh. *Nutrients,* 12.

BRAC 2015. Alive & Thrive's integration of Maternal Nutrition Interventions into BRAC MNCH (rural) Programme in Bangladesh: Process Documentation of the preparatory activities. Final Report based on the field-testing of MN intervention package at Muktagacha, Mymensingh. BRAC Health, Nutrition and Population Program. Dhaka, Bangladesh

Clemmons, L. & Griffiths, M. 2016. Maternal nutrition: Findings and recommendations from consultations and TIPs with women and their families in Amhara, Oromia and SNNP regions. Power point presentation. Manoff Group, for Feed the Future, USAID.gov

CMS 2015. Center for Media Studies (CMS). Nutrition Practices in Uttar Pradesh. Formative Research Report. <https://www.aliveandthrive.org/sites/default/files/attachments/UP_Formative-research_02-Feb.pdf>. Washington DC: Alive & Thrive.

Hirvonen, K. & Wolle, A. 2019. Consumption, production, market access and affordability of nutritious foods in the SNNP Region of Ethiopia. Addis Ababa, Ethiopia. Washington DC: Alive & Thrive and International Food Policy Research Institute.

Kachwaha, S., Nguyen, P. H., DeFreese, M., Avula, R., Cyriac, S., Girard, A. & Menon, P. 2020. Assessing the Economic Feasibility of Assuring Nutritionally Adequate Diets for Vulnerable Populations in Uttar Pradesh, India: Findings from a "Cost of the Diet" Analysis. *Curr Dev Nutr,* 4**,** nzaa169.

Kim, S., Ouédraogo, C., Zagré, R., Sununtnasuk, C., Ganaba, R. & Menon, P. 2020a. Assessing the Feasibility of Integrating a Package of Maternal Nutrition Interventions into ANC Services in Burkina Faso. Baseline Survey Report. Washington, D.C: Alive & Thrive.

Kim, S., Sununtnasuk, C., Faas, S., Tadesse, A. & Menon, P. 2020b. A Feasibility Study of Integrating Maternal Nutrition Interventions into ANC Services in Ethiopia: A Cluster-Randomized Evaluation. Baseline Survey Report. Washington, D.C: Alive & Thrive.

Ky-Zerbo, R. O., Sanou, K. & Kere, S. 2019. Recherche formative pour la mise en œuvre d’un paquet d’interventions en nutrition maternelle a travers les soins prenatals au Burkina Faso. Unpublished report.

Nguyen, P. H., Kachwaha, S., Avula, R., Young, M., Tran, L. M., Ghosh, S., Agrawal, R., Escobar-Alegria, J., Patil, S. & Menon, P. 2019. Maternal nutrition practices in Uttar Pradesh, India: Role of key influential demand and supply factors. *Matern Child Nutr,* 15**,** e12839.

Nguyen, P. H., Kachwaha, S., Tran, L. M., Mani, S., Avula, R., Raj, N., Patil, S. & Menon, P. 2018. Integrating Maternal Nutrition Interventions in Existing Reproductive, Maternal, Newborn and Child Health Services in Uttar Pradesh, India. Alive & Thrive Baseline Survey Report. Washington, D.C: Alive & Thrive.

Nguyen, P. H., Sanghvi, T., Kim, S. S., Tran, L. M., Afsana, K., Mahmud, Z., Aktar, B. & Menon, P. 2017. Factors influencing maternal nutrition practices in a large scale maternal, newborn and child health program in Bangladesh. *PLoS One,* 12**,** e0179873.

Nguyen, P. H., Tran, L. M., Rawat, R. & Menon, P. 2015. Assessing the Operational Feasibility of Strengthening Maternal Nutrition Interventions in Maternal, Neonatal, and Child Health Platform in Bangladesh. Alive & Thrive Baseline Survey Report. Washington, D.C: Alive & Thrive.

PMA2020 2018. PMA2020 Nutrition Survey Results: Burkina Faso.

Schuler, S. 2015. Formative Research on Maternal Nutrition, Bangladesh. Alive &Thrive, Bangladesh.

Sosthène, K. C. 2020. Enquêtes auprès des femmes enceintes à l’issue d’une SPN et des femmes ayant accouchés au CSPS dans les 3 jours après l’accouchement dans 20 CSPS de deux régions du Burkina Faso: Hauts Bassins et Boucle de Mouhoun. Report on second round of exit interviews. CERTIS for Alive and Thrive. .

Young, M. F., Bootwala, A., Kachwaha, S., Avula, R., Ghosh, S., Sharma, P. K., Shastri, V. D., Forissier, T., Menon, P. & Nguyen, P. H. 2021. Understanding Implementation and Improving Nutrition Interventions: Barriers and Facilitators of Using Data Strategically to Inform the Implementation of Maternal Nutrition in Uttar Pradesh, India. *Curr Dev Nutr,* 5**,** nzab081.

Zongo, F., Soubiego, A., Some, J. & Lougue, M. 2019. Assessment of the Supply Chain for Iron and Folic Acid and other products related to the Prevention of Maternal Anemia in Two Regions of Burkina Faso. Unpublished report, A&T/CERTIS, Ouagadougou.
